# Progress in palliative care in Israel: comparative mapping and next steps

**DOI:** 10.1186/2045-4015-1-9

**Published:** 2012-02-20

**Authors:** Netta Bentur, Linda L Emanuel, Nathan Cherney

**Affiliations:** 1 Center for Research on Aging, Myers-JDC-Brookdale Institute, Jerusalem, Israel; 2 Buehler Center on Aging, Health & Society, Feinberg School of Medicine, Chicago, USA; 3 Cancer Pain & Palliative Medicine Unit in the Dept. of Medical Oncology, Shaare Zedek Medical Center, Jerusalem, Israel

## Abstract

Palliative care was established rapidly in some countries, while in other countries its establishment has taken a different trajectory. This paper identifies core steps in developing a medical specialty and examines those taken by Israel as compared with the US and England for palliative care. It considers the next steps Israel may take.

Palliative care aims to provide quality of life for those with serious illnesses by attending to the illness-prompted physical, mental, social, and spiritual needs of patients and their families. It has ancient roots in medicine; its modern iteration began against the backdrop of new cures and life-sustaining technology which challenged conceptions of how to respect the sanctity of life.

The first modern hospice was created by Saunders; it provided proof that palliative care works, and this has occurred in Israel as well (the first step). Another key step is usually skills development among clinicians; in Israel, few education and training opportunities exist so far. Specialty recognition also has not yet occurred in Israel. Service development remains limited and a major shortage of services exists, compared to the US. Research capacity in Israel is also limited. Policy to develop and sustain palliative care in Israel is underway; in 2009, the Ministry of Health established policy for implementing palliative care. However, it still lacks a financially viable infrastructure.

We conclude that palliative care in Israel is emerging but has far to go. Adequate resource allocation, educational guidelines, credentialed manpower and specialty leadership are the key factors that palliative care development in Israel needs.

## Introduction

The profession of medicine is in constant evolution. With each new development, the health service delivery infrastructure, healthcare policies, clinical providers, and patient population all need to respond in order to implement the development. During the decades of rapidly occurring scientific breakthroughs, translation of discovery into technology for use in existing specialty practices was the main form of development and adaptation. Occasionally, however, a new specialty is created. This is the case for palliative care.

### Palliative Care as a Specialty

Ironically, palliation of suffering is perhaps the most ancient and essential feature of medicine. During the scientific era of the twentieth century, those goals were underemphasized. Following the recognition by Cicely Saunders [1] and soon after by Balfour Mount, that patients' humanity was getting lost in the quest for a cure and the incurable were dying without comfort or closure, the practice of modern hospice and palliative care was born. Initially developed for the misunderstood and often abandoned population of the dying, hospice started in England as a type of care that occurred outside traditional healthcare settings, in care homes where people could go to die in comfort, while in the US, hospice primarily focused on providing a similar type of care in the patient's home. With Balfour Mount's efforts in Canada, palliative care was conceived as the same type of care but it was seen as necessarily integrated with mainstream medical services. Some three decades later, the term "palliative care" refers largely to palliation for anyone who needs it, no matter their prognosis; and the term "hospice" tends to refer to end-of-life care. Suffering was defined by Cicely Saunders as occurring in four overlapping domains of human experience: physical, psychological, social, and spiritual [2]. Palliative care, whether in hospice or mainstream care settings, was defined in various ways, but the definitions quite consistently addressed management of suffering in all these domains.

As pain and other symptom management became scientifically complex and required skills as demanding as in any recognized specialty, in country after country, palliative care began to be recognized as a specialty alongside cardiology, infectious disease and other mainstream specialties. Today, palliative care's integration and development are not complete, but its relatively rapid establishment has nonetheless been impressive. Different countries have taken different trajectories. Today, in the US, palliative care's integration and development are not complete, but its relatively rapid establishment has nonetheless been impressive. This paper examines the trajectory taken by Israel, to date, as compared with that in the US, and considers the next steps Israel may take.

### Steps in Capacity Development for Palliative Care

Capacity development for a new specialty is not a stereotypical process. Nonetheless, some core steps are likely to be identified and addressed by each country as it manages its development. Scholars have described such developments in conceptual and social science terms [3]; here we simply identify some of the steps that we feel are critical within the context of a comparative description of progress in palliative care in Israel and the US.

A new specialty's history is likely to start with the recognition of a need. Then a pioneer program demonstrates that the need can be met. With this "proof of concept," or demonstration of efficacy and feasibility in one setting, the core content of what that specialty offers can be defined and brought as a new skill-set to practitioners. Many barriers must be overcome just to reach this stage, but the next challenges are also great. In order for the skill-set to be taken up and used, not only the culture of medicine but also the culture of the population must be receptive to and desirous of the services. These developments need to be matched with new policies that support the services. With increased use of services, quality control becomes necessary; a specialty association with standards, protocols and norms, and formal specialty standing within the medical profession, with specialist provider accreditation, is the traditional setting for quality control. This provides the necessary support for creation of specialty training programs, development of an adequate number of providers in the discipline, and dissemination of core skills to all providers, so that the services can be made available to all who need them.

To further the field, the establishment of research resources and research skills suitable to the subject matter among professionals then becomes possible and necessary. Policy that supports the services and research may then be supported and refined by evidence. Later stages in the development of a mature field include integration of the specialty services with other services, ensuring access to and use by as full a population of those who need the services as possible, and finally, sustained continuous quality improvement. These steps [4] are listed below and are described below with a focus on Israel's development of palliative care against the backdrop of its evolution in other countries, especially the US, ending with recommendations for Israel's next steps for the establishment of palliative care as an integrated service within the institution of medicine. These steps and their order are not intended as absolute requirements and it is reasonable to assume that the optimal path differs with country.

Archetypal steps in the development of a new specialty in medicine

1. A need is perceived

2. An entity establishes that the need can be effectively met (proof of concept)

3. The core subject matter of the area is defined

4. Skills to match the subject matter are developed for the workforce

5. The culture of medicine and the population served want the services

6. Specialty associations form and formal bodies recognize the area as a specialty

7. Capacity is developed through disseminated training and local service units

8. Research capacity to continue the specialty's advancement is developed

9. Policy to support the service delivery and research is created

10. Maturation of the services develops, scaling and establishing quality control

#### 1. Perceived Need

Awareness of the needs of the dying took shape in the setting of emerging life-sustaining technology in the 1970s and with the discovery that it is possible to exist in a persistent vegetative state. The first country that began to talk about taking back control of the dying process out of the hands of medicine was the UK, where Dame Cicely Saunders founded the St Christopher Hospice in London in 1967; that was the first modern hospice [5]. Many Western countries, including the US, embraced the idea of hospice care. In 1969, Florence Wald, who is accredited with bringing the hospice movement to the United States, invited Saunders to the US to learn from her experience [6]. Grassroots efforts to develop hospice care further during the 1970s led to a Health Care Financing Administration demonstration project at the end of the decade, and the passage of the Medicare Hospice Benefit by the US Congress in 1982 [7].

In the US, the same concerns resulted in advocacy for living wills to legally direct physicians to withhold or withdraw unwanted life-sustaining technology. In 1990, the Patient Self Determination Act (OBRA**-90**, Pub. L. 101-508, 104 Stat. 1388, enacted on November 5, 1990) required that all patients be asked if they have such determinations. A parallel movement went further, demanding the right to assisted suicide or euthanasia. Eventually, the United States Supreme Court recognized the right of an individual to refuse unwanted medical treatment, leaving the creation of laws concerning assisted suicide and euthanasia to the individual states. Patient and family experience in hospice care was so dramatically better than their experience of traditional hospital care that, by the time New York passed the Palliative Care Information Act in 2010 (Chapter 331, section 2997-c of the New York Laws of 2010) [8] requiring that all terminal patients be informed of hospice and palliative care options, public perception had already established hospice care as the culturally expected standard of care for the terminally ill.

In Israel, awareness of the individual's rights regarding end-of-life decisions exists, but there are cultural concerns that impede its development into reality and policy. For many Jews in Israel the concept of "sanctity of life" (*kedushat hakhayim*) is a central value [9-11]; this contributes to a tendency for patients to request and for physicians to provide "aggressive" modes of care even as patients approach the end of life. The community is pluralistic and substantial segments of the public and health professionals reject this outlook and would prefer end-of-life practices found in other countries. Consequently, health providers in Israel face the challenge of respecting personal autonomy and supplying quality end-of-life care, while also taking traditional values into account. This situation is further complicated by a lack of widespread familiarity with palliative care precepts among many healthcare professionals. Indeed, among the public, many fear that, in the terminal stages of their lives, they will receive more medical care and less pain relief than they want [12].

Responding to prolonged public pressure, the government has initiated efforts to tackle end-of-life issues. In 2000, Israel's Minister of Health established a public committee on the sensitive issue of end-of-life care. The committee was chaired by Rabbi Avraham Steinberg - who is also an ethicist and professor of medicine - and included representatives of different Jewish denominations, the larger minority groups within Israel, physicians, philosophers and ethicists. In 2005, the Dying Patient Act was enacted in Israel [13].

In 2004, the director-general of the Ministry of Health appointed a professional committee to formulate recommendations for setting guidelines for palliative care in Israel. The committee was headed by Prof. Pesach Schwartzman and included experts from the national councils for community health, oncology, palliative care, geriatrics and children's health. In 2009, the director-general of the Ministry of Health issued a directive policy statement describing standards for the development and provision of palliative care services for hospitals and the health plans.

#### 2. Proof of Concept

A long history of pre-modern hospice care in Europe and the Holy Land dates back to the Middle Ages. Hospices were religiously motivated charitable places that gave care to the sick and dying - mostly along the Crusader and pilgrims paths [14]. Modern hospice is very different; indeed, its only common theme with past hospices is care for the dying. Palliative care is defined by the World Health Organization as follows:

Palliative care is an approach that improves the quality of life of patients and their families facing the problem associated with life-threatening illness, through the prevention and relief of suffering by means of early identification and impeccable assessment and treatment of pain and other problems, physical, psychosocial and spiritual. Palliative care provides relief from pain and other distressing symptoms; affirms life and regards dying as a normal process; intends neither to hasten or postpone death; integrates the psychological and spiritual aspects of patient care; offers a support system to help patients live as actively as possible until death; offers a support system to help the family cope during the patients illness and in their own bereavement; uses a team approach to address the needs of patients and their families, including bereavement counseling, if indicated; will enhance quality of life, and may also positively influence the course of illness; is applicable early in the course of illness, in conjunction with other therapies that are intended to prolong life, such as chemotherapy or radiation therapy; and includes those investigations needed to better understand and manage distressing clinical complications. http://www.who.int/cancer/palliative/definition/en/.

The above-mentioned first hospice, started by Saunders, provided proof that the hospice and palliative care concept could be used. The overwhelming satisfaction of patients and families served by her freestanding hospice proved that the concept was not only feasible but also desirable. Like-minded institutions made the case in other countries. One was opened in Connecticut in the US [6] and another, opened soon after, became the first to take patients in Chicago, Illinois. In 1975, one was established at the St Luke's Hospital Center in New York: It incorporated into an existing medical center an array of services ranging from inpatient care, home care, clinic care, to bereavement counseling for families. All were successful and established the integrated care that Saunders envisioned, as did the Medicare hospice benefit, which required that care be provided in both the home and inpatient settings, and included psychosocial care, spiritual care, and bereavement care alongside medicine and nursing as required services.

Interest in establishing the efficacy and effectiveness of the model in Israel began about three decades ago. In 1981, the head of the Geriatric department at Sheba Medical Center in Tel Hashomer, Dr. Israel Abraham Adunski, traveled to England after hearing of the work of Cicely Saunders and was duly impressed. Following the visit, he established a committee to discuss the possibility of developing adequate services for the terminally ill and their families. With the help of the Israel Cancer Association, this led to the establishment of the first inpatient hospice in Israel, in 1983, and in 1986, the second inpatient hospice opened in Jerusalem, at Hadassah, Mount Scopus [15].

By the early 1980s, recognition and awareness of palliative care needs led to the first community homecare service with a component of palliative care, which was run by nurses funded and trained by the Israel Cancer Association. In 1978, these nurses developed postgraduate specialist oncology training that included a component of palliative care and symptom management. This created a viable option for terminal patients wishing to be cared for at home. First, the nurses worked as part of the family physician system, and later as part of homecare and home-hospice community health teams. During the late 1980s and the 1990s, seven home-hospice units were opened all over the country, supplying palliative care for cancer patients. However, they dealt almost exclusively with cancer patients and did not provide for patients with chronic illnesses [16,17].

In 1983, the first home hospice for adult cancer patients run by volunteers was funded in Kiryat Tivon [18] and in 1994, a nonprofit home hospice was founded by Prof. Nancy Caroline, in northern Israel, in order to provide palliative care support for the families of patients from all sectors of society - Jews, Moslems, Christians, Druze and other groups. In an effort to provide palliative care services to another remote area, a Bedouin mobile palliative care unit was established in the Negev desert, in 2004, in order to reach isolated places [19].

In 1994, the Shaare Zedek Medical Center established the first cancer center fully integrating oncology and palliative care services, under the leadership of Prof. Raphael Catane and Dr. Nathan Cherny [20]. This initiative was followed by the development of similar services in the cancer centers at the Rambam Medical Center in 2003, and at the Davidson Cancer Center in 2008. More limited programs exist in other medical centers. For example, in 2002 an ambulatory palliative service was implemented in Soroka University Medical Center including both an adult oncology department and a pediatric hematoncology department that provide hospital-based adult and pediatric palliative care consultation and support at the regional hospital in southern Israel [21].

The Israeli Association of Palliative Care (*Tmicha*/IAPC) was established in 1993, as a voluntary organization for all health professionals, trained volunteers, and interested lay people who identify with its philosophy and are actively involved in palliative care in Israel. It is active in encouraging professional and public education and in promoting the development of services. The association places emphasis on the importance of multidisciplinary collaboration between health professionals, produces a quarterly newsletter and organizes an annual professional conference. The Israel Palliative Medical Society (IPMS) was established in 1996, as a branch of the Israel Medical Association. The aim of the IPMS is to represent physicians practicing palliative medicine and to promote palliative medical services, education and research. Both Tmicha and the IPMS are members of the European Association for Palliative Care (EAPC) [22,23].

In the mid-2000s, the UJA-Federation of New York introduced the field of spiritual care and chaplaincy in Israel, in order to provide a comprehensive and holistic response, similar to the work being done in New York. In early 2006, it launched a major new funding initiative for the development and provision of spiritual care services and training programs in Israel [24,25]. The first chaplaincy program was established at the Shaare Zedek Medical Center in 2002, and shortly thereafter, a chaplaincy-training program was launched with the support of the UJA-Federation of New York. Subsequently, a network of chaplaincy and spiritual care providers was established.

#### 3. Core Content Defined

Guidelines for defining the core content of the field of palliative and hospice care were initially guided by the Oxford Textbook of Palliative Care from England. Funding by the Robert Wood Johnson Foundation in the 1990s of a series of professional education programs - for nurses, physicians (non-palliative care specialist) and specialty fellows - provided the vehicle against which to define the core content of palliative care. The curriculum contents were decided by expert consensus groups from across the US [4]. This was followed by the National Guidelines project and several US textbooks [26,27].

In 2005, the Israeli Ministry of Health commissioned an expert report to define core content of palliative care services. This report was supplemented by additional inputs from the Society of Palliative Medicine Physicians. Based on these reports and submissions, in July 2009 the director-general of the Ministry of Health issued a policy statement describing minimal standards for the development of palliative care services for hospitals and for the provision of home palliative care by the health plans [28].

#### 4. Skill Development among Clinical Providers

Skill development among clinicians, nurses and other healthcare professionals is essential in order to disseminate palliative services. Without proper training, there is no prospect of furthering the provision and access of services. In the US, core competencies were provided over a 10-year period by the above-mentioned education dissemination projects, and then by post-graduate specialty training for physicians and nurses [4]. Currently, there are about 60 palliative care fellowships in the US. Palliative care specialists must have satisfactorily completed a year of full-time clinical care in a fellowship program and pass the American Board of Medical Specialties' hospice and palliative medicine written exam.

In Israel, there are only a few frameworks that offer education and in-service training on the subject. All programs that have existed have been temporary and funded by donations. The curricula of training programs for health professionals (both undergraduate and postgraduate) provide very little formal training in palliative care. Issues relating to suffering and terminal illness have been included in the curricula of medical and nursing schools only as part of general topics such as ethical issues or pain management [29,30]. Courses providing more comprehensive training existed but were discretionary, and were offered only in hospitals or in training programs in which there were particularly motivated educators and/or clinicians.

In an attempt to meet the needs of health professionals for palliative knowledge and skills, a program was launched in 2005 - a collaboration between the UJA Federation of New York, ESHEL (the Association for Planning and Developing Services for the Aged in Israel), Ben-Gurion University of the Negev, and the "Support" organization (a multi-disciplinary organization for palliative care in Israel). The aim was to develop and implement a national, interdisciplinary training program in palliative care, for physicians, nurses, and social workers. This would increase the awareness and knowledge of healthcare professionals in the principles of palliative care and expand the scope and quality of palliative care provided to patients and their families throughout Israel. The courses comprise six sessions, follow a uniform 43-hour curriculum, and are carried out all over the county, in hospitals, health plans and a few nursing homes [31,32]. To date, more than 1,200 participants have taken part - about two-thirds of them nurses, a sixth social workers, and a sixth physicians, as well as a small number of professionals from other fields. Since the UJA funding expired, the ongoing costs have been covered by the health plans, hospitals and nursing homes.

The external educational departments in the universities invite professionals to participate in enrichment and continuing medical education courses in which they can earn points toward receiving continuing education grants. These courses are similar to the American CME only in that they entitle participants to receive a grant in addition to their salary. (In Israel, professionals, including doctors, are not yet required to prove that they have participated in the continuing educational courses or to take repeat licensing tests.) The courses are not funded by the universities and therefore the participants are required to pay for them.

These were the main courses:

a. Tel Aviv Medical School: Palliative Medicine - This one-year program, established in 1998, includes some 300 hours of teaching and seminars. Although ostensibly for physicians, this course has also included nurses and pharmacists (not every year; this year it is not taking place).

b. Social Sciences Faculty in Tel Aviv University - Enrichment courses for gerontologists: four meetings on the topic of the right to quality.

c. The Ben-Gurion University Medical School with the Israel Association for Prevention of Pain - Pain management course [33].

Unfortunately, most of these programs were term limited. Other education and training programs for professionals caring for patients with life-threatening diseases or the elderly exist, but they are not systematic or integrated into the education systems.

#### 5. Cultural Alignment

The US culture of youth and a sense of invulnerability fostered a culture of death denial such that, until the 1980s, care near the end of life was neglected. With the advent of the persistent vegetative state, the US culture started to examine old and new realities of dying. The movement to control dying by planning in advance with documentation of wishes began in the 1970s, and developed in parallel with the euthanasia movement [34]. Although small pockets in the US, starting with Oregon, passed legislation to legalize physician-assisted suicide, the main outcome of the effort nationally was to recognized the right of an individual to refuse unwanted medical treatment (Washington v. Glucksberg, 117 S.Ct. 2258 1997, Vacco v. Quill, 117 S.Ct. 2293 1997). With the aging of society and the burdens of the "sandwich generation" (struggling financially to care for their aging parents while providing for their high-school and college-age children) increasing in the 2000s, people began to seek realistic solutions to aging and dying. Today, hospice and palliative care are household words and part of the normative expectations of many or most citizens who are seeking quality care for the seriously ill and dying.

The relationship between religion and state is a crucial issue in culture, identity and political life in Israeli society, including palliative care [35]. Judaism is a religion of laws pertaining to everyday life [36]. The sanctity of human life (*kedushat hakhayim*) is a central value in Israeli culture and human life is deemed of infinite value. These beliefs were echoed in the results of a study that compared physicians' attitudes in Israel and the United States towards end-of-life decisions. There were more respondents in Israel than in the US (72% vs. 57%) who said their professional duty was foremost to preserve the patient's life [37]. Nonetheless, Judaism also recognizes that all life is finite and therefore is compatible with the philosophy of palliative and hospice care. Relief of suffering is deemed important. The process of dying must be respected when it is clearly occurring, imminent, and irreversible [38]. Although most Jewish people do not strictly adhere to Jewish law, these tenets do impact on beliefs and culture. In Israel today, there is a desire to merge cultural identity and heritage with democratic and liberal values. These issues were addressed in the committee chaired by Professor Steinberg, which found a solution that resolved the tension between the right of individual autonomy and Israeli cultural heritage [13]. People with diverse beliefs and values were able to bridge the gap between individual autonomy, death with dignity, and avoidance of prolonged suffering.

Almost no empirical data are available in Israel concerning the knowledge, attitudes and perceptions of the public regarding palliative care, and there is limited information on the knowledge and perceptions of general practitioners and specialists. This was confirmed in the results of a qualitative study that examined the Israeli family physicians' attitudes towards the Dying Patient Act, which found that very few physicians discussed end-of-life care or the option of advance care planning with their patients. Although the physicians are aware of the option of preparing an advanced directive or of appointing a healthcare proxy for end-of-life issues, they are concerned about cultural barriers to implementing the act [39].

There are no robust statistics on how many people are referred to palliative care; however, considering the limited and patchy availability of services and the misconceptions that abound, overall referral is low. For many, palliative and hospice care are perceived to be the "end of the road" or "the last stop." Some patients and their families are skeptical about palliative care even when it is offered to them, and some even refuse the services. However, in those institutions or regions with well-developed local programs, there are reports of very high resource utilization.

In recent years, several television and newspaper stories have followed the "struggle" of cancer patients, mostly children. Although some of them died, in most cases, aspects relating to their end-of-life care were not described. With few notable "positive" exceptions, the popular press rarely offers stories of people facing end-of-life situations. One recent well-known example is Mr. Dov Lautman, an industrialist and philanthropist, who is frequently interviewed in the press or television about how he is facing ongoing neurological functional deterioration of his situation because of ALS and is, at the same time, continuing his philanthropic activities.

#### 6. Specialty Recognition

The American Board of Medical Specialties in the US recognized hospice and palliative medicine as a discipline in 2006. Education had been proceeding prior to that so that a number of fellowship programs were available for specialty training in the area already, and board certification had been offered by the American Board of Hospice and Palliative Medicine. After specialty recognition, compliance with standard regulations for supervised training was adopted.

Specialty recognition in Israel is determined by the Scientific Committee of the Israel Medical Association. Several attempts have thus far been made to apply for specialty recognition by the Association of Palliative Medicine Physicians within the Israel Medical Association and through the Israel Association of Palliative Care. A substantial limiting factor that has precluded progress has been the strict regulatory requirement that there be at least two fully trained mentors in each training institution, and furthermore that there be more than one training institution in the country. A further application is pending. The Scientific Council has presented the possibility of developing a fellowship program as a stepping stone to the later development of an independent specialty, once the obstacles of manpower thresholds are overcome.

In 2009, an injunction was signed recognizing palliative care as a specialty for nurses [40]. This action brought an end to the long struggle led by Dr Shoshana Riba, Head Nurse and Chief Nursing Officer to create a new professional status for nurses in Israel. Veteran palliative care nurses who have undergone advanced training in various settings have been granted recognition as clinical nurse specialists Two Israeli universities are currently developing formal academic programs and graduates will be required to undergo licensing examinations [41].

#### 7. Service Provision

Hospices exist in the US that are freestanding, non-profit, freestanding for profit, chain for profit, and associated with larger health organizations. Little is known about differences, if any, in quality between the types. A study that used a nationally representative data set of hospices surveyed in the National Home and Hospice Care Survey, 1992-2000, found Medicare hospice certification was associated with a significantly broader range of services provided to patients and it was significantly more pronounced among for-profit hospices than among nonprofit [42].

Palliative care services that combine this type of care with traditional care in many disciplines are also increasingly available: A hospital-based consulting service usually provides care at the request of the attending physician from wherever the patient is receiving his or her predominant care - cardiology, oncology, surgery, etc. Some of these palliative care services have dedicated beds and their own service for patients for whom palliative care becomes the primary type of care. These dedicated beds are served exclusively by palliative care physicians, nurses and other disciplinary members of the palliative care team, and non-palliative care clinicians take on a consulting role. Other, usually smaller, palliative care services provide their care wherever the patient is, without the option of moving to a dedicated palliative care service bed. Much of the successful dissemination and development of service capacity in the US was due to the funding of the Center to Advance Palliative Care, and the finding by researchers of empirical data consistent with a robust business case allowed its integration to move forward rapidly against the backdrop of spiraling healthcare costs [43,44]. The implementation of the Affordable Care Act of April 2010 is still emerging http://www.healthreform.gov/index1.html, but increased funding for home-based care may improve use of palliative care as well.

Israel's Ministry of Health has declared that palliative and hospice services are covered under the basket of services that every health plan should supply to their enrollees according to the National Health Insurance Law [28]. Indeed, the law stipulates that the health plans have to provide their members with all necessary services within the framework of a mandated benefits package. However, certain issues and services are not sufficiently defined or clarified by the law: The law does not include detailed eligibility criteria for palliative care, nor does it provide formulation of the process of determining eligibility, and there are no directives on how to decide which services will be permitted to refer a patient for palliative or hospice care. The Law does not determine the framework for the provision of this care, the types of care that people are eligible to receive, the frequency and scope of care, or the professions and education of those providing palliative care.

Hospital- and community-based palliative and hospice services do exist in Israel, but to a very limited extent. As of 2010, few hospitals provide consultative or inpatient palliative care services. Three hospitals - the Shaare Zedek Medical Center in Jerusalem, Rambam Medical Center in Haifa, and the Belinson Medical Center in Petach Tikva - each have well developed palliative care services for cancer patients that have met all the criteria for the ESMO designation in integrated oncology and palliative care. These services include consultation services, ambulatory palliative care, inpatient palliative care, and close correlation with community resources for home care. Others hospitals have more limited services, with the availability of either a part-time palliative care consultant physician or nurse. Most of the 15 cancer centers in Israel do not have any designated physicians responsible for palliative care services.

There are three inpatient hospices in Israel - in Jerusalem, Tel Aviv, and Haifa - which have been allotted a total of about 80 beds, and serve some 1,000 patients per year. Additionally, 3-4 hospitals belonging to the Christian Mission take in end-of-life patients who cannot be at home but have not yet reached the hospice stage, or for whom there is no hospice in their vicinity. They offer dedicated care, but the staff's palliative training is very limited.

There are about 8 home-hospice units all over the country. They rely on multiple sources of financing, such as Clalit's Health Plan (the biggest of Israel's four health plans) budgets, the sale of services to other health plans, and mainly philanthropic contributions. Consequently, there is no steady supply of funds to these units, making their existence precarious, and impeding their ability to expand their activities.

Only a few of the physicians working in the home-hospice units have formal training in palliative care, but almost all of the nurses have studied more than a basic level of oncology and/or palliative care in Israel or abroad. The home-hospice units differ significantly in their patterns of care. Each unit has established procedures that govern the number of regular physician and nurse visits. The staff is usually available 24 hours a day, in person and by telephone. About two-thirds of home-hospice patients are treated simultaneously by other services - primarily by community clinics and oncology services in hospitals.

Overall, opioid availability and accessibility for cancer patients is good. A wide range of opioids is available for the management of moderate and strong cancer pain. Morphine, oxycodone and transdermal fentanyl are widely used. There is less use of parenteral hydromorphone and oral methadone, though both are available. Transmucosal fentanyl is available to patients who have not had adequate relief with oral immediate release formulations or in situations where oral medication is contraindicated. For cancer patients, medications covered in the non-discretionary basket of services, which include all opioids, are dispensed at no cost.

Prescriptions do not require any special forms and need not be in duplicate or triplicate. They must include personal details, including identification number and address, and the prescription must be written both in numbers and in longhand. If a patient presents with a prescription that has a technical error, pharmacists have little or no discretion to honor or correct the prescription and the patient must return with a fully valid prescription. In general, prescriptions are valid for a 10-day supply of medication. However, if there is an annotation that the patient suffers chronic cancer pain, prescription for a 30-day supply is permitted. Most opioids are widely available in community pharmacies and patients need not present themselves to special pharmacies. The regulations regarding opioid prescription and dispensing make no provision for emergency physician prescriptions by telephone or fax, or emergency prescriptions by nurses or by pharmacists.

Israel saw a modest increase in opioid consumption in the years 2000-2008 and a rapid increase per capita in opioid consumption during the former decade. Data for the 2000-2008 period (for all treatment settings, private and public) show that consumption of the five strong opioids (requiring a special prescription form) increased by 47%, from 2.46 DDD/1,000 inhabitants per day in 2000 to 3.61 DDD/1,000 inhabitants per day in 2008. This has been associated with substantial changes in the pattern of differential opioid prescribing, characterized by increased prescription of oxycodone, fentanyl, buprenorphine, and dextropropoxyphene, and decreased prescription of morphine, pethidine, and codeine [20,45]. A seven-year follow-up study indicated an increase in opioid prescriptions for cancer and non-cancer pain with an annual increase in OME per capita. During the study period, there were no relevant changes in regulations and policy [46].

In addition to the previously described palliative care services they have already committed to, Israel's four health plans operate home medical care units in all of their districts, which provide medical, nursing, and rehabilitative home care. These units treat housebound individuals, mostly elderly, who suffer from a variety of chronic and functional disabilities. The units supply palliative care for about 5,000 patients with metastasized cancer and neurologic and degenerative diseases per year. However, they almost never provide palliative care for dying dementia patients. Generally, staff of these services are available during working hours only. Although some are on call by telephone until evening, with some exceptions they are not generally available to provide services in the evening and at night.

The palliative care credentials of members of these teams are very varied, and some have no formal training in palliative care. However, most of the homecare units employ oncology nurses on staff, who are available by telephone 24 hours a day. These oncology/palliative care nurses are credentialed and have received oncological and/or palliative training in Israel or abroad. For the most part, these nurses guide other community healthcare providers in coping with complex situations and sometimes care directly for dying patients. They play a central role in coordinating hospital and community services, developing and implementing oncology and palliative projects in their district, training medical personnel, and overseeing this type of care in their district [47].

#### 8. Research Capacity Development

In the US, funding for research in palliative care began with the National Cancer Institute, an institute within the National Institutes for Health - the main government funding agency for medical research. Its primary purpose is funding for cancer research, and palliative care has been included, in recognition that palliative care is a part of comprehensive cancer care. The American Cancer Society research arm followed suit and has provided research funding for palliative care as well. Most recently, the National Palliative Care Research Center was created as a part of the Center to Advance Palliative Care, with the mission to provide grant support to researchers and career development awards to incoming researchers in palliative care. Institutes for other areas of medical care, such as the National Heart Lung and Blood Institute, are increasingly aware of the need to support research in palliative care for their population of patients.

There are several research groups in Israel that are involved in conducting clinical and health services research in the area of palliative care, in order to improve services provided to patients and their families. These include researchers at Ben-Gurion University of the Negev, the Shaare Zedek Medical Center in Jerusalem, and the Myers-JDC-Brookdale Institute in Jerusalem. In addition to clinical topics, the issues examined include the improvement of service organization and options for their expansion, training needs, quality of care, and the cost of providing palliative services to end-of-life patients compared to the cost of non-palliative care.

Most of the research grants in Israel, whether from academic institutions, the Ministry of Health, or the National Institute for Health Policy and Health Services Research, are not earmarked for a specific area. Palliative researchers must compete with researchers in other areas that are sometimes considered more important or more urgent. Indeed, studies on the quality of care for end-of life-patients have often been shelved after receiving low marks with regard to their importance.

There are no organizations that focus on funding research on palliative care in Israel and, similarly, there has been no effort to develop researchers with suitable career tracks and training to do the research.

The palliative care organization in Israel have not initiated any multi centers studies and they have no framework for collaborative research. Individual researchers, however, have been productive and have published substantially and Israeli specialists and researchers are involved in the Expert Working Group of the Steering Committee of the Research Network of the European Association of Palliative Care, that revised and updated the guidelines on the use of morphine in the management of cancer pain [48,49], the use of sedation in palliative care [50], and other topics.

So far, palliative research has focused predominantly on cancer patients with much less research addressing palliative care for other chronic conditions.

#### 9. Policy

To become available, hospice care needed a mechanism of reimbursement for its services, on a par with other medical services. In 1979, the Health Care Financing Administration began demonstration projects with 26 hospices. By 1986, the Medicare (the US medical benefit for the elderly) Hospice Benefit, which provides services to all with a prognosis of 6 months or less to live, was made permanent. Some states also provide hospice to Medicaid (the US medical benefit for the poor) eligible patients [51].

In addition to reimbursement policies, information has been essential in the uptake of service use. The (above-described) 1990 Patient Self-Determination Act (that requires healthcare organizations to ask patients about advance care planning and to conduct community outreach regarding advance care planning) and several state laws that provide for the use of transportable Physician Orders for Life Sustaining Treatment (POLST) [52] or that require provision of information about hospice and palliative care to terminal patients (see above-noted New York Palliative Care Information Act), have aided awareness and use of hospice and palliative care services in the US.

In Israel, the Dying Patient Act has been enacted. The law reflected an actual, tangible need among healthcare professionals and the Israeli public. The most distinguishing feature of the Dying Patient Act was the attempt to respect cultural reluctance to withdraw treatment while offering a practical solution that respects the wishes of patients and families, and allows patients to end their lives with dignity. The law supported the right of terminally ill patients with a life expectancy of less than 6 months to formulate advance directives that may include the forgoing of treatments and the withdrawal of ventilator supports. The Orthodox population's objections to the withdrawal of ventilators were mitigated by the proposal of changing ventilator support to an intermittent treatment by virtue of timers which would allow for the non-initiation of a further cycle of therapy. The decision to end life support could be made by not resetting the timer rather than active termination, satisfying the Jewish law that prohibits the active deed of ending one's life. The infrastructure for this solution is only now, 5 years after the legislation, in a testing phase and it has not yet been implemented. Presently, there are no statistics concerning the number of individuals filling out Advance Directives documents. According to an interview with the Chief Officer of Medical Ethics at the Ministry of Health, in charge of implementing the Dying Patient Act, they have received approximately 2,000 advance directive documents to be approved and entered into a database.

The director-general adopted the policy paper that was submitted by the committee in 2005. The policy paper adopted the definition of the WHO, according to which palliative care is a therapeutic approach aimed at improving the quality of life of patients coping with incurable diseases and their families, by preventing and relieving suffering via identification and careful evaluation of symptoms, by management of pain, and by dealing with other issues, such as physical, mental and spiritual problems. In mid-2009, after a long period of debates and discussions, the director-general of the Ministry of Health published a policy statement for the implementation of palliative care in the healthcare system. The circular defines the target populations for palliative care services as patients with considerable physical and emotional distress at any disease stage, including terminally ill cancer patients, patients with end-stage heart failure, patients with end-stage lung disease, patients with end-stage liver failure, patients with end-stage renal failure, patients with severe stroke, patients with advanced neurodegenerative disease, and unconscious patients. It defines minimal standards for palliative care services, which must include a physician, a nurse, a psychologist and a social worker. The document also details credentialing, and hours of availability. According to the directive, the health plans, the general hospitals, and long-term care institutions, have to make all arrangements required for provision of these services within four years of the issue of the directive, and have to report to the Head of Medical Administration on their progress towards service implementation within one year of the publication of the directions. This initiative was not accompanied by budgetary expansion specific to this purpose.

#### 10. Maturation: Scaling Up and Quality Control

In the US, increasing citizen and professional awareness and increasing service availability have driven up the utilization of hospice and palliative care considerably over the last decade. For 2009, NHPCO estimates that approximately 41.6% of all deaths in the United States were under the care of a hospice program [53]. A great majority of health delivery organizations now provide, or are affiliated with organizations that provide, palliative and hospice care [54]. The Joint Commission (previously the Joint Commission on Accreditation of Healthcare Organizations or JCAHO) has provided, since 1984, voluntary accreditation to hospices and palliative care services. As with other healthcare organizations and services, they periodically and randomly visit any entity that seeks their accreditation. Evaluating the state of services by examining facilities and records and by interviewing providers, they discern whether their criteria for quality are met and provide their seal of accreditation, or not, accordingly. More generally, the medical system in the US is increasingly aware that research and adoption of accreditation standards are not enough to improve practice standards. The combination of research in palliative care with the emerging field of dissemination and implementation research will be necessary [55].

In Israel, hospital- and community-based palliative and hospice services exist, but it is estimated they serve less than 10-15% of those who could benefit from them. However, as noted above, there are no robust statistics on how many people are referred to palliative care, but considering the lack of services and the misconceptions that abound, referral is probably very low.

Although a large proportion of the residents of long-term care institutions suffer from multiple symptoms at the end of life, most of these institutions abide by conservative treatment methods, which consist primarily of medication to alleviate pain and other physical symptoms. A few geriatric hospitals and institutions have recently begun implementing the hospice approach, by providing psychological and emotional support while treating physical symptoms.

There is no sustained quality assessment and improvement and/or any regulatory agency, voluntary or mandated, that has formulated a set of criteria to be met, that assesses the quality of services, that gives accreditation to the palliative service organization, or refuses the accreditation depending on whether or not the service organization meets its criteria.

In January 2011, the Ministry of Health started to assess implementation of the ministerial directive by health plans and hospitals. It will also verify whether organizations have formed a multidisciplinary team to supply palliative services, and if appropriate training was provided.

### Next Steps for Palliative Care in Israel

Tracking the development of palliative care according to the archetypal steps we defined, reveals that the belief that every individual has the right to palliative care at the end of life is gaining ground in Israel. Israel has clearly made significant progress, yet palliative services have not been widely accepted as a fully integrated service in the nation's healthcare delivery system, and therefore further steps are needed.

As professional skill development is essential in order to develop, implement and disseminate palliative services, there needs to be significant expansion in education and training in this field. There is no standard curriculum for current programs that offer palliative education at graduate level in medical and nursing schools. There is a need to construct a palliative care curriculum - a modular and dynamic model for teaching medical students, residents, fellows, practicing doctors and other health professionals. In addition, the development and implementation of an obligatory palliative training program is necessary for students in all health professions, and for other professionals dealing with elderly and chronic patients.

In the post-graduate framework there are few programs that offer education and in-service training on the subject. While the basic courses that have been developed by Ben-Gurion University and ESHEL, and the advanced leaning program at Tel Aviv University have merit, they do not suffice for comprehensive advanced training and credentialing. The formal development of an educational pathway for specialist nurses in palliative care is a major and important development.

Advanced training for physicians remains a problem, particularly with the lack of cooperation from the scientific committee of the Israel Medical Association to recognize palliative medicine as a medical specialty. The existing palliative care professional organizations, Tmicha and the IPMS, should consider the approach of the American Society for Hospice and Palliative Medicine, which initiated its own internal accrediting system before achieving formal specialist recognition. By developing its own training requirements and credentialing framework, it would essentially provide responsible quality control as well as providing a ready framework for implementation if and when specialty or subspecialty recognition is achieved. Once center, Shaare Zedek Medical center, is providing a 2-year supervised fellowship position.

It is especially important to both require palliative care training for at least part of the staff in nursing homes, and to expand palliative care services through primary physicians with an emphasis on symptom management.

Achieving these educational goals requires both financial resources and trained manpower. In order to train and educate a sufficient cadre of doctors, nurses and other medical staff, it is unlikely that the system can continue to rely on donations and temporary funds, as has been done until now. As part of the efforts to develop the discipline, it will be necessary to divert resources toward the education and training of professional manpower, in order to create specialists in palliative care who can in turn teach and train a large cadre of people, and in order to deepen and expand the curriculum in the few on-the-job training programs currently running.

As described above, in order to build on current policy, in July 2009, the Israeli Ministry of Health published a policy directive requiring universal access to palliative care within a few years. There is concern that success of this initiative may be limited by the failure to specifically relate to standards of care, standards of assessment, and quality of care of this new endeavor [44].

Under-resourcing of palliative care is a substantial problem. Ministry of Health policy initiatives for the development and provision of new palliative care services and training, without allocation of any new resources for this purpose, may not produce substantial change. This concern is especially grave given the extreme limitation of resources already allotted to the healthcare sector, which is struggling to address other core elements of its mandate.

The Ministry would do well to reconsider and re-assess this decision, for a number of reasons:

1. The basket of services mandated by the National Health Insurance Law is based on the basket of services supplied by the Clalit health plan in 1994, which at that time did not provide any palliative services.

2. Since the law was passed nearly two decades ago, there has been great development and an increase in palliative care and hospice services in all Western countries. This increase is reflected in the number and types of patients, illnesses and diagnoses, as well as the types of services offered. In the US, for example, this is the service that has enjoyed the greatest increase in funding and resource allocation in the past decade. Consequently, it is unrealistic to expect that these services be developed and supplied in Israel in the required amount and quality, without providing monetary remuneration to the suppliers. Hospitals and health plans, and particularly nursing facilities, are already functioning with minimal budgets. It is unreasonable to expect that they develop high-quality palliative services without receiving separate funding for expended and new services. There is very real concern that, in order to comply with the Ministry of Health's demands, they will develop palliative services of poor quality, unsatisfactory professional standard and insufficient scope.

3. Studies in Israel and abroad, have shown that palliative services are more cost-effective than other medical services for similar patients, without detracting from the quality of care and the quality of their end of life. It is therefore possible that investing resources in the development and implementation of palliative services could save much greater expenditures in the future.

A relevant issue, currently hotly debated in Israel, has to do with equity in the availability and accessibility of health services [56,57]. Research has recently revealed large gaps in the scope and quality of Israeli medical services between the major population centers and the less densely populated outlying regions in the north and south of the country, as well as gaps between population groups with different socio-economic characteristics. Thus, it is important to ensure that palliative services be established and supplied throughout the country, and in particular in the periphery and in places already suffering a lack of services.

Another issue that should be addressed is the awareness and attitude of the Israeli public (both Jewish and Arab) concerning the role of palliative care to improve quality of life and to ensure the provision of comfort in death and support for the family. Addressing these issues should be predicated on credible data regarding currently existing attitudes and barriers to the utilization of palliative care services.

## Conclusions

Palliative care services in Israel are substantially less well developed than in the United States. The development of grass-root programs, the existing infrastructure of primary home care, processional organization and focal areas of great expertise, provide excellent substrata for the development of palliative care in Israel. Although policy reforms are important and laudable, they are not enough. Inadequate resource allocation, lack of educational guidelines at all levels of medical and paramedical training, failure to facilitate the development of credentialed manpower, and failure to develop specialty level leadership, are among the key factors that continue to thwart the development of palliative care in Israel.

## Competing interests

The authors declare that they have no competing interests.

## Authors' contributions

NB and NC wrote sections pertaining to Israel. LE wrote sections pertaining to the United States. All authors developed together the steps in capacity development for palliative care, and read and approved the final manuscript.

## Authors' information

Netta Bentur is a Senior Researcher at the Myers-JDC Brookdale Institute in Jerusalem, and an Associate Professor at Tel-Aviv University's Stanley Steyer School for Health Professionals.

Linda L. Emanuel is Director of the Buehler Center on Aging, Health & Society at Northwestern University, where she also holds appointments as a professor in the medical school and an adjunct professor at the school of management.

Nathan Cherny MBBS, FRACP, FRCP is the Norman Levan Chair of Humanistic Medicine at Share Zedek Medical Center and an Associate Professor in Internal Medicine at Ben Gurion University. He is also the director of the Cancer Pain and Palliative Medicine Service in the Department of Medical Oncology at the Shaare Zedek Medical Center, Jerusalem, Israel.
